# The first coordination compound of deprotonated 2-bromo­nicotinic acid: crystal structure of a dinuclear paddle-wheel copper(II) complex

**DOI:** 10.1107/S2056989020000390

**Published:** 2020-01-17

**Authors:** Nives Politeo, Mateja Pisačić, Marijana Đaković, Vesna Sokol, Boris-Marko Kukovec

**Affiliations:** a Department of Physical Chemistry, Faculty of Chemistry and Technology, University of Split, Ruđera Boškovića 35, HR-21000 Split, Croatia; b Department of Chemistry, Faculty of Science, University of Zagreb, Horvatovac 102a, HR-10000 Zagreb, Croatia

**Keywords:** crystal structure, copper(II), 2-bromo­nicotinic acid, dinuclear paddle-wheel cluster, hydrogen-bond motif

## Abstract

A copper(II) dimer with the deprotonated anion of 2-bromo­nicotinic acid (2-BrnicH), namely, tetrakis(μ-2-bromonicotinato-κ^2^ *O*:*O*′)bis[aquacopper(­II)](*Cu*—*Cu*), [Cu_2_(H_2_O)_2_(C_6_H_3_BrNO_2_)_4_] or [Cu_2_(H_2_O)_2_(2-Brnic)_4_], (**1**), was prepared by the reaction of copper(II) chloride dihydrate and 2-bromo­nicotinic acid in water. The copper(II) ion in **1** has a distorted square-pyramidal coordination environment, achieved by four carboxyl­ate O atoms in the basal plane and the water mol­ecule in the apical position. The pair of symmetry-related copper(II) ions are connected into a centrosymmetric paddle-wheel dinuclear cluster [Cu⋯Cu = 2.6470 (11) Å] *via* four *O*,*O*′-bridging 2-bromo­nicotinate ligands in the *syn*-*syn* coordination mode. In the extended structure of **1**, the cluster mol­ecules are assembled into an infinite two-dimensional hydrogen-bonded network lying parallel to the (001) plane *via* strong O—H⋯O and O—H⋯N hydrogen bonds, leading to the formation of various hydrogen-bond ring motifs: dimeric *R* ^2^_2_(8) and *R* ^2^_2_(16) loops and a tetra­meric *R* ^4^_4_(16) loop. The Hirshfeld surface analysis was also performed in order to better illustrate the nature and abundance of the inter­molecular contacts in the structure of **1**.

## Abstract 2

The copper(II) ion has a distorted square-pyramidal coordination environment, achieved by four carboxyl­ate O atoms in the basal plane and the water mol­ecule in the apical position. The pair of symmetry-related copper(II) ions are connected into a centrosymmetric paddle-wheel dinuclear cluster *via* four *O*,*O*′-bridging 2-bromo­nicotinate ligands. In the extended structure, the cluster mol­ecules are assembled into an infinite two-dimensional hydrogen-bonded network lying parallel to the (001) plane *via* strong O—H⋯O and O—H⋯N hydrogen bonds, leading to the formation of various hydrogen-bond ring motifs: dimeric 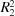
(8) and 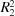
(16) loops and a tetra­meric 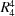
(16) loop.

## Chemical context   

Copper(II) carboxyl­ates have been studied extensively because of their structural diversity and related possible applications. The origin of this diversity is in the variable donating ability of the carboxyl­ate oxygen atoms and in the nature of the other coordinated ligands (Iqbal *et al.*, 2013 ▸; Song *et al.*, 2009 ▸). The structural diversity of copper(II) carboxyl­ates depends strongly on the inclusion of additional coligands (*e.g.* hy­droxy, alk­oxy and azide ions), which are able to mediate magnetic coupling between the copper(II) ions and to enable ferromagnetic and anti­ferromagnetic inter­actions *via* their various bridging modes (Zhang *et al.*, 2012 ▸; Ma *et al.*, 2014 ▸).

Copper(II) carboxyl­ates can find applications as biologic­ally active agents (Fountoulaki *et al.*, 2011 ▸; Lim *et al.*, 2009 ▸), as electrochemical (Bharathi *et al.*, 2006 ▸; Bharathi *et al.*, 2007 ▸), luminescent (Mei *et al.*, 2016 ▸) and magnetic materials (Rigamonti *et al.*, 2013 ▸; Cejudo *et al.*, 2002 ▸; Colacio *et al.*, 1999 ▸) and in the construction of MOFs. Copper(II) carboxyl­ates can exhibit various magnetic properties – from the expected paramagnetic behavior (due to the *d* ^9^ electronic configuration of the metal ion) to ferromagnetic and anti­ferromagnetic behavior, depending on the ligand coordination modes and copper(II) coordination environments (Rigamonti *et al.*, 2013 ▸; Cejudo *et al.*, 2002 ▸; Colacio *et al.*, 1999 ▸). The metal–metal inter­action in copper(II) carboxyl­ates is also an important factor that can affect their magnetic properties and the structure (Rigamonti *et al.*, 2013 ▸; Cejudo *et al.*, 2002 ▸; Colacio *et al.*. 1999 ▸; Ozarowski *et al.*, 2015 ▸; Poppl *et al.*, 2008 ▸; Sarma *et al.*, 2008 ▸).

Polynuclear copper(II) carboxyl­ates have gained much inter­est in recent years (Zhu *et al.*, 2010 ▸; Zhang *et al.*, 2010 ▸; Sheikh *et al.*, 2013 ▸), for example the copper(II) metal–organic framework containing benzene-1,3,5-tri­carboxyl­ate (HKUST-1) is based on dinuclear paddle-wheel copper(II) moieties, with inter­esting magnetic properties (Chui *et al.*, 1999 ▸; Pichon *et al.*, 2007 ▸; Furukawa *et al.*, 2008 ▸). These paddle-wheel copper(II) moieties have frequently been used in the design of coordination polymers and MOFs as secondary building units (SBU) (Baca *et al.*, 2008 ▸; Roubeau & Clerac, 2008 ▸; Bai *et al.*, 2008 ▸).

Nicotinic acid has been widely used as a complexing agent for various metal ions and many crystal structures of its metal complexes (almost 900) have been reported and deposited in the Cambridge Structural Database (CSD, Version 5.40, searched October 2019; Groom *et al.*, 2016 ▸). However, metal complexes of nicotinic acid derivatives have been much less explored. For example, no metal complexes of 2-bromo­nicotinic acid (2-BrnicH) have been reported so far.

Our goal was to prepare 2-bromo­nicotinate copper(II) complexes for the above-mentioned significance of copper(II) carboxyl­ates. The syntheses were carried out in aqueous solution to ensure that water mol­ecules (either coordinated and/or hydrated) would be present in their crystal structures, enabling the formation of hydrogen-bonded frameworks. Furthermore, we wanted to explore the type and occurrence of hydrogen bond motifs within the obtained frameworks.
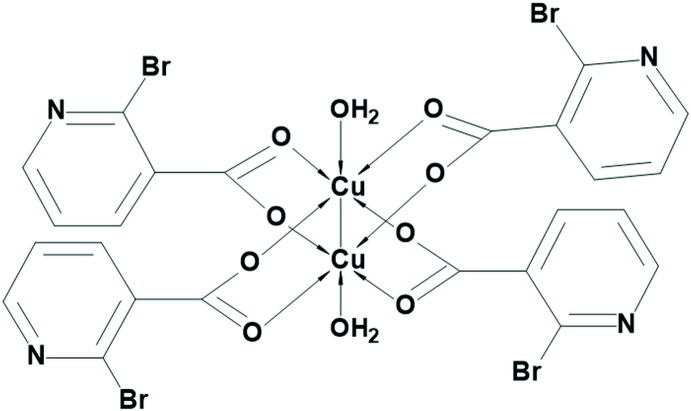


In this work, we report the synthesis and characterization of the first metal complex with 2-bromo­nicotinic acid – a dinuclear paddle-wheel copper(II) cluster, [Cu(H_2_O)(2-Brnic)_2_]_2_ (**1**). Although similar dinuclear paddle-wheel copper(II) carboxyl­ates with coordinated water mol­ecules in the axial positions are well-established and have been extensively studied, there are only a few examples of such compounds that are analogous to **1**, containing nicotinate derivatives [2-chloro­nicotinate, 2-meth­oxy­nicotinate, 2-eth­oxy­nicotinate and 2-(naphthalen-2-yl­methyl­sulfan­yl)nico­tin­ate] as carboxyl­ates (Moncol *et al.*, 2007 ▸; Jun, Lu *et al.*, 2013 ▸; Jun, Wei-Ping *et al.*, 2013 ▸; Adhikari *et al.*, 2016 ▸).

## Structural commentary   

**Figure 1:**
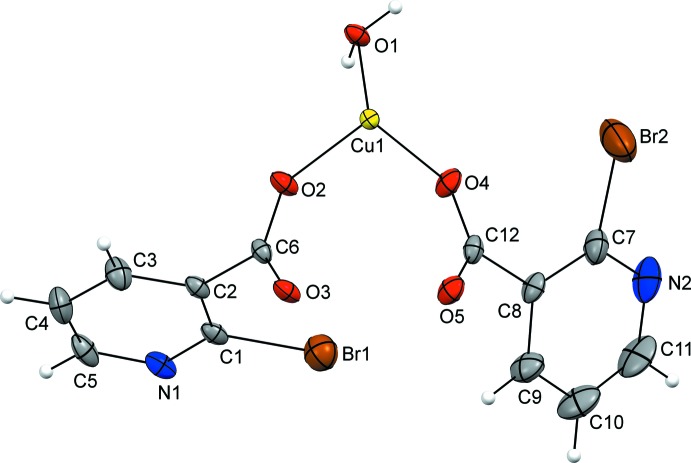
The asymmetric unit of **1**, with the atomic numbering scheme. The displacement ellipsoids are drawn at the 40% probability level.

**Figure 2:**
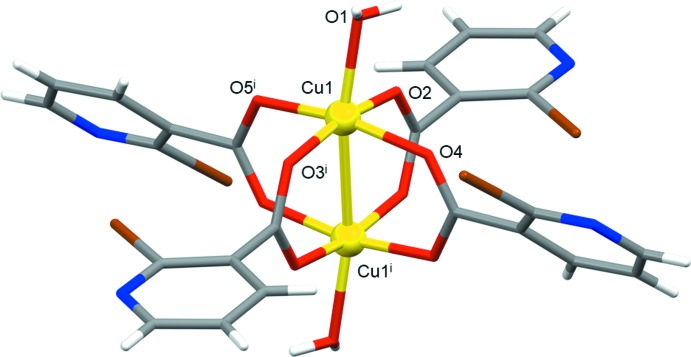
The dinuclear cluster of **1** with selected atoms labeled [symmetry code: (i) −*x* + 1, −*y* + 2, −*z* + 1].

The asymmetric unit of **1** consists of a copper(II) ion coordinated by a water mol­ecule and by two deprotonated *O*-monodentate 2-bromo­nicotinate ligands (Fig. 1 ▸). The coordination environment of the copper(II) ion can be described as a distorted square pyramid as τ amounts to 0 [τ = (α − β) / 60° (α and β are the largest angles), τ = 0 for an ideal square pyramid and 1 for an ideal trigonal bipyramid; Addison *et al.*, 1984 ▸]. The basal plane of the pyramid is defined by four carboxyl­ate O atoms [O2, O4, O3^i^ and O5^i^; symmetry code: (i) −*x* + 1, −*y* + 2, −*z* + 1] from four 2-bromo­nicotinate ligands while its apical position is occupied by the aqua atom O1 (Fig. 2 ▸). The two symmetry-related copper(II) ions are connected into a centrosymmetric paddle-wheel dinuclear cluster [with a Cu⋯Cu contact length of 2.6470 (11) Å] *via* four *O*,*O*′-bridging 2-bromo­nicotinate ligands in the *syn*–*syn* coordination mode (Phetmung & Nucharoen, 2019 ▸). This Cu⋯Cu inter­action is slightly longer than the sum of the covalent radii of Cu atoms (2.64 Å; Cordero *et al.*, 2008 ▸). The Cu⋯Cu contact length in **1** is also somewhat longer than those in related paddle-wheel copper(II) clusters with nicotinic acid derivatives (Jun, Lu *et al.*, 2013 ▸; Jun, Wei-Ping *et al.*, 2013 ▸; Adhikari *et al.*, 2016 ▸), but almost equal to that seen in the paddle-wheel copper(II) cluster with 2-chloro­nicotinic acid (Moncol *et al.*, 2007 ▸). The Cu—O_c_ and Cu—O_w_ (c = carboxyl­ate, w = water) bond lengths are comparable with literature values (Moncol *et al.*, 2007 ▸; Jun, Lu *et al.*, 2013 ▸; Jun, Wei-Ping *et al.*, 2013 ▸; Adhikari *et al.*, 2016 ▸).

The copper(II) ion in **1** is situated nearly at the center of the basal plane with an out-of-plane deviation of 0.209 (2) Å in the direction of the apical Cu1—O1 bond (Fig. 2 ▸). The square-pyramidal coordination environment around the copper(II) ion is distorted, as indicated by the angles for the *cis* [88.95 (17)–103.05 (15)°] and *trans* [167.76 (17)–167.80 (15)°] pairs of ligating atoms. There is also a small tetra­gonal elongation because of the Jahn–Teller effect: Cu1—O1 [2.120 (4)] is somewhat longer than the other four Cu—O bond lengths [1.950 (4)–1.979 (4) Å].

## Supra­molecular features   

**Figure 3:**
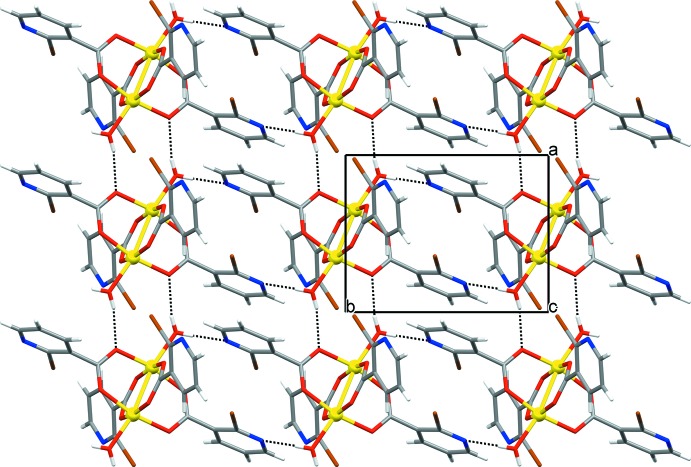
A fragment of the infinite two-dimensional hydrogen-bonded network of **1** viewed along the *c* axis. The cluster mol­ecules are connected by O—H⋯O and O—H⋯N hydrogen bonds (represented by the dotted lines) within the network.

The extended structure of **1** features strong O—H⋯O and O—H⋯N hydrogen bonds, weak C—H⋯O hydrogen bonds (Table 1 ▸) and anion–π inter­actions [C1—Br1⋯*Cg*1; where *Cg*1 is the centroid of the pyridine ring N1/C1–C5; Br1⋯*Cg*1 = 3.629 (2) Å; C1—Br1⋯*Cg*1 = 103.27 (17)°]. The strong hydrogen bonds link the cluster mol­ecules into an infinite two-dimensional hydrogen-bonded network lying parallel to the (001) plane (Fig. 3 ▸), with the anion–π inter­actions consolidating the layered network. The layers are assembled into a three-dimensional network by the C—H⋯O bonds.

**Figure 4:**
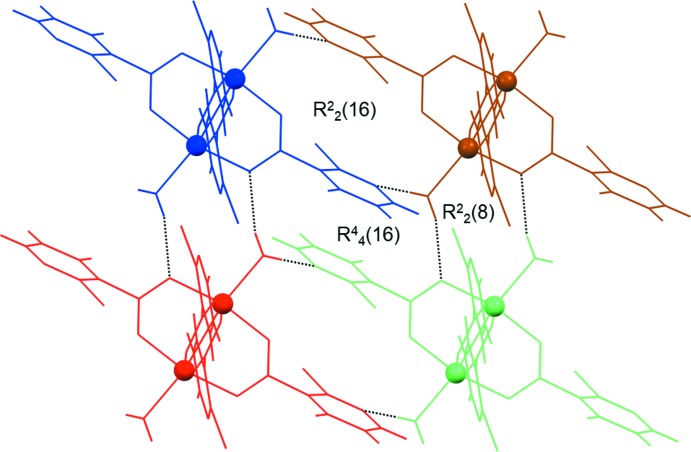
The distinctive hydrogen-bonded ring motifs (represented by dotted lines) found within the layered network of **1**, *viz*. the dimeric 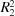
(8) and 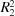
(16) motifs and the tetra­meric 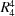
(16) motif. The various symmetry-related cluster mol­ecules are shown in blue, brown, red and green (see text).

There are some distinctive hydrogen-bonded ring motifs within the layered network of **1** (Fig. 4 ▸). The dimeric 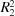
(8) motif is formed between symmetry-related mol­ecules (indicated in brown and green) *via* two water mol­ecules and two carboxyl­ate O atoms, the dimeric 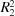
(16) motif is formed between symmetry-related mol­ecules (indicated in brown and blue) *via* two water mol­ecules and two pyridine N atoms, while the tetra­meric 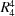
(16) motif is formed by symmetry-related mol­ecules (indicated in blue, brown, red and green) *via* two water mol­ecules and two pyridine N atoms (Fig. 4 ▸). The water mol­ecules participate in the formation of motifs as both single- and double-proton donors [single in the 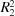
(8) and 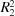
(16) motifs and double in the 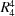
(16) motif], while the carboxyl­ate O and pyridine N atoms participate as single-proton acceptors exclusively. These hydrogen-bonded motifs in **1** are quite different from those in the crystal structures of related paddle-wheel copper(II) clusters with nicotinate derivatives (Jun, Lu *et al.*, 2013 ▸; Jun, Wei-Ping *et al.*, 2013 ▸; Adhikari *et al.*, 2016 ▸). This difference is not surprising in the case of copper(II) clusters with 2-eth­oxy­nicotinate and 2-(naphthalen-2-yl­methyl­sulfan­yl)nicotinate because the presence of water mol­ecules of crystallization drastically affects the crystal packing (Jun, Wei-Ping *et al.*, 2013 ▸; Adhikari *et al.*, 2016 ▸). The difference in the hydrogen-bonded ring motifs in the case of the copper(II) cluster with 2-meth­oxy­nicotinate (Jun, Lu *et al.*, 2013 ▸) can be attributed to the different supra­molecular arrangement of the cluster mol­ecules, which are connected into a hydrogen-bonded chain, as opposed to a hydrogen-bonded network in the case of **1**. Furthermore, it seems that the substituents in the nicotinate derivatives (meth­oxy group *versus* bromine atom) have a great influence on the supra­molecular assemblies and on the hydrogen-bond motif types in the respective crystal packings because of the difference in the proton-acceptor abilities of the two substituents.

## Hirshfeld surface analysis   

**Figure 5:**
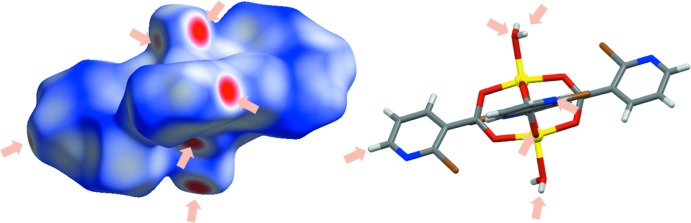
Hirshfeld surfaces on the mol­ecule of **1**. Regions highlighted in red represent shorter contacts, while longer contacts are blue.

**Figure 6:**
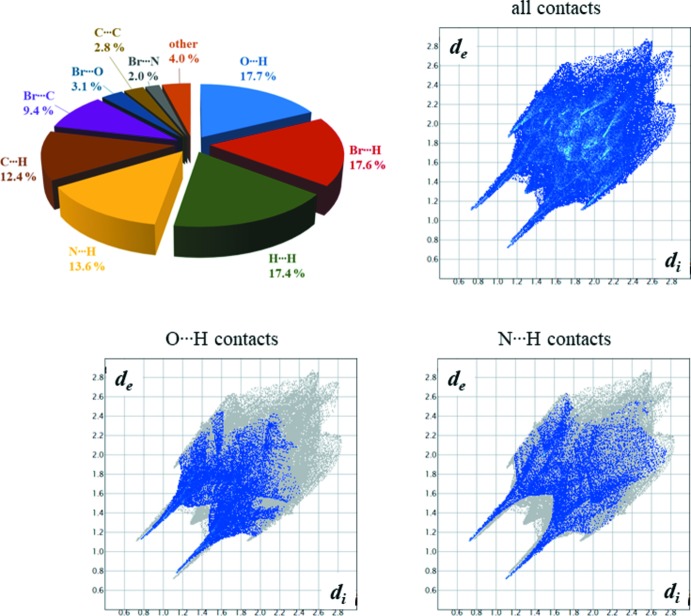
The fingerprint plots showing distances from each point on the Hirshfeld surface to the nearest atom inside (*d*_i_) and outside (*d*_e_), presented for all contacts and for contributions of O—H⋯O and O—H⋯N hydrogen bonds in **1**. The percentage contributions of all other selected contacts are shown in a pie chart.

The Hirshfeld surface analysis of **1** was performed using *CrystalExplorer17.5* (Wolff *et al.*, 2012 ▸). Normalized contact distances, *d*_norm_, were plotted with standard color settings: regions highlighted in red represent shorter contacts, while longer contacts are shown in blue (Fig. 5 ▸). The fingerprint plots show distances from each point on the Hirshfeld surface to the nearest atom inside (*d*_i_) and outside (*d*_e_), and are presented for all contacts and for the contributions of two primary contacts, O—H⋯O and O—H⋯N hydrogen bonds (Fig. 6 ▸). The percentage contributions of all other selected contacts are presented as a pie chart (Fig. 6 ▸).

## PXRD and thermal analysis   

The PXRD analysis was used to confirm the bulk content of **1** (see Fig. S1 in the supporting information). The experimental and calculated PXRD traces of **1** are in very good agreement, confirming the phase purity of **1**.

The thermal stability of **1**, as determined from the TG curve, is up to 140°C (Fig. S2 in the supporting information). The two coordinated water mol­ecules (observed mass loss 3.9%, calculated 3.7%) were released at 176°C (endothermic peak at the DSC curve). The thermal decomposition of **1** continues *via* two consecutive steps (observed mass losses 10.6% and 24.7%) in the temperature range of 190–390°C (exothermic peak at 195°C), which corresponds to the release of approximately one and a half 2-bromo­nicotinate ligands (calculated mass loss 31.2%). The decomposition finishes with the release of another two 2-bromo­nicotinate ligands (observed mass loss 46.7%, calculated 41.6%) in the final step (temperature range of 390–600°C). The observed residue (13.3%) at 600°C, remained after total decomposition of **1**, corresponds to CuO. The experimental mass fraction of copper (10.7%) matches nicely with the calculated mass fraction (13.1%).

## Materials and methods   

All chemicals for the synthesis were purchased from commercial sources (Merck) and used as received without further purification. The IR spectrum was obtained in the range 4000–400 cm^−1^ on a Perkin–Elmer Spectrum Two^TM^ FTIR-spectrometer in the ATR mode. The PXRD trace was recorded on a Philips PW 1850 diffractometer, Cu *K*α radiation, voltage 40 kV, current 40 mA, in the angle range 5–50° (2*θ*) with a step size of 0.02°. Simultaneous TGA/DSC measurements were performed at a heating rate of 10°C min^−1^ in the temperature range 25–800°C, under an oxygen flow of 50 mL min^−1^ on an Mettler–Toledo TGA/DSC 3+ instrument. Approximately 2 mg of the sample was placed in a standard alumina crucible (70 µl).

## Synthesis and crystallization   

2-Bromo­nicotinic acid (0.0502 g; 0.2485 mmol) was dissolved in distilled water (5 ml) with the addition of a drop of concentrated ammonia solution and then mixed and stirred with an aqueous copper(II) chloride dihydrate solution (0.0220 g; 0.1290 mmol in 2 ml of distilled water). The pH of the obtained solution was adjusted to 6–7 by adding an ammonia solution dropwise. The clear solution was left to evaporate slowly at room temperature for a month until blue crystals of **1**, suitable for X-ray diffraction measurements, were obtained, which were collected by filtration, washed with ethanol and dried *in vacuo*. Yield: 0.0209 g (17%). Selected IR bands (ATR) (*ν*, cm^−1^): 3432 [ν(O—H)], 3065 [*ν*(C—H)], 1623[*ν*(C=O)), 1385 [*ν*(C—N)_pyridine_] (Fig. S3, Table S1 in the supporting information).

## Refinement   

Crystal data, data collection and structure refinement details are summarized in Table 2 ▸. The C-bound H atoms were placed geometrically (C—H = 0.93 Å) and refined as riding atoms. The water-mol­ecule H atoms were found in difference-Fourier maps and refined with the O—H distances restrained to an average value of 0.82 Å using DFIX and DANG instructions. The constraint *U*_iso_(H) = 1.2*U*_eq_(carrier) was applied in all cases. The highest difference peak is 1.00 Å away from Br2 and the deepest difference hole is 0.78 Å away from the same atom.

## Supplementary Material

Crystal structure: contains datablock(s) I. DOI: 10.1107/S2056989020000390/hb7877sup1.cif

Structure factors: contains datablock(s) I. DOI: 10.1107/S2056989020000390/hb7877Isup2.hkl

PXRD, TG-DSC, IR data. DOI: 10.1107/S2056989020000390/hb7877sup3.docx

CCDC reference: 1977430

Additional supporting information:  crystallographic information; 3D view; checkCIF report

## Figures and Tables

**Table 1:** Hydrogen-bond geometry (Å, °)

*D*—H⋯*A*	*D*—H	H⋯*A*	*D*⋯*A*	*D*—H⋯*A*
O1—H11⋯O3^i^	0.82 (1)	2.07 (2)	2.868 (5)	165 (6)
O1—H12⋯N1^ii^	0.82 (1)	2.02 (2)	2.816 (6)	166 (6)
C11—H11*A*⋯O1^iii^	0.93	2.51	3.403 (8)	161

Symmetry codes: (i) 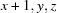; (ii) 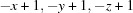; (iii) 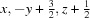.

**Table 2:** Experimental details

Crystal data	
Chemical formula	[Cu(H_2_O)(C_6_H_3_BrNO_2_)_2_]_2_
*M*_r_	967.13
Crystal system, space group	Monoclinic, *P*2_1_/*c*
Temperature (K)	296
*a*, *b*, *c* (Å)	7.5596 (2), 9.7402 (3), 20.5332 (7)
β (°)	94.345 (3)
*V* (Å^3^)	1507.56 (8)
*Z*	2
Radiation type	Mo *K*α
μ (mm^−1^)	6.77
Crystal size (mm)	0.59 × 0.42 × 0.33

Data collection	
Diffractometer	Oxford Diffraction Xcalibur2 diffractometer with Sapphire 3 CCD detector
Absorption correction	Multi-scan (*CrysAlis PRO*; Rigaku, 2018 ▸)
*T*_min_, *T*_max_	0.288, 1.000
No. of measured, independent and observed [*I* > 2σ(*I*)] reflections	19205, 2622, 2392
*R*_int_	0.028
(sin θ/λ)_max_ (Å^−1^)	0.595

Refinement	
*R*[*F* ^2^ > 2σ(*F* ^2^)], *wR*(*F* ^2^), *S*	0.050, 0.134, 1.06
No. of reflections	2622
No. of parameters	205
No. of restraints	3
H-atom treatment	H atoms treated by a mixture of independent and constrained refinement
	
Δρ_max_, Δρ_min_ (e Å^−3^)	1.34, −2.06

Computer programs: *CrysAlis PRO* (Rigaku, 2018 ▸), *SHELXT* (Sheldrick, 2015*a* ▸), *SHELXL2018/3* (Sheldrick, 2015*b* ▸) and *Mercury* (Macrae *et al.*, 2008 ▸).

## supplementary crystallographic information

### Crystal data

**Table:**

[Cu_2_(C_6_H_3_BrNO_2_)_4_(H_2_O)_2_]	*F*(000) = 932
*M**_r_* = 967.13	*D*_x_ = 2.131 Mg m^−^^3^
Monoclinic, *P*2_1_/*c*	Mo *K*α radiation, λ = 0.71073 Å
*a* = 7.5596 (2) Å	Cell parameters from 7938 reflections
*b* = 9.7402 (3) Å	θ = 4.6–32.5°
*c* = 20.5332 (7) Å	µ = 6.77 mm^−^^1^
β = 94.345 (3)°	*T* = 296 K
*V* = 1507.56 (8) Å^3^	Prism, green–blue
*Z* = 2	0.59 × 0.42 × 0.33 mm

### Data collection

**Table:**

Oxford Diffraction Xcalibur2 diffractometer with Sapphire 3 CCD detector	2392 reflections with *I* > 2σ(*I*)
ω–scan	*R*_int_ = 0.028
Absorption correction: multi-scan (CrysAlisPro; Rigaku, 2018)	θ_max_ = 25.0°, θ_min_ = 4.3°
*T*_min_ = 0.288, *T*_max_ = 1.000	*h* = −8→8
19205 measured reflections	*k* = −11→11
2622 independent reflections	*l* = −24→24

### Refinement

**Table:**

Refinement on *F*^2^	Primary atom site location: dual
Least-squares matrix: full	Hydrogen site location: mixed
*R*[*F*^2^ > 2σ(*F*^2^)] = 0.050	H atoms treated by a mixture of independent and constrained refinement
*wR*(*F*^2^) = 0.134	*w* = 1/[σ^2^(*F*_o_^2^) + (0.0666*P*)^2^ + 10.4972*P*] where *P* = (*F*_o_^2^ + 2*F*_c_^2^)/3
*S* = 1.06	(Δ/σ)_max_ = 0.001
2622 reflections	Δρ_max_ = 1.34 e Å^−^^3^
205 parameters	Δρ_min_ = −2.06 e Å^−^^3^
3 restraints	

### Special details

**Table:**

Geometry. All esds (except the esd in the dihedral angle between two l.s. planes) are estimated using the full covariance matrix. The cell esds are taken into account individually in the estimation of esds in distances, angles and torsion angles; correlations between esds in cell parameters are only used when they are defined by crystal symmetry. An approximate (isotropic) treatment of cell esds is used for estimating esds involving l.s. planes.

### Fractional atomic coordinates and isotropic or equivalent isotropic displacement parameters (Å^2^)

**Table:**

	*x*	*y*	*z*	*U*_iso_*/*U*_eq_	
Cu1	0.65135 (8)	0.95254 (6)	0.48054 (3)	0.0136 (2)	
Br1	0.35523 (10)	0.55987 (8)	0.58074 (4)	0.0462 (2)	
Br2	0.97277 (15)	0.95629 (13)	0.67690 (5)	0.0838 (4)	
N1	0.1831 (6)	0.4145 (5)	0.4818 (3)	0.0259 (10)	
N2	0.8306 (9)	0.8063 (7)	0.7703 (3)	0.0518 (16)	
O1	0.8705 (5)	0.8353 (4)	0.45349 (18)	0.0201 (8)	
H11	0.969 (4)	0.857 (5)	0.469 (3)	0.024*	
H12	0.852 (7)	0.758 (3)	0.466 (3)	0.024*	
O2	0.4953 (5)	0.7928 (4)	0.4598 (2)	0.0251 (8)	
O3	0.2397 (5)	0.8692 (4)	0.49343 (19)	0.0232 (8)	
O4	0.6771 (5)	0.8938 (4)	0.57194 (17)	0.0265 (9)	
O5	0.4213 (5)	0.9715 (4)	0.60518 (18)	0.0278 (9)	
C1	0.2511 (7)	0.5362 (5)	0.4956 (3)	0.0214 (11)	
C2	0.2524 (6)	0.6442 (5)	0.4517 (3)	0.0192 (11)	
C3	0.1765 (8)	0.6213 (6)	0.3893 (3)	0.0318 (13)	
H3	0.172615	0.691086	0.358327	0.038*	
C4	0.1065 (9)	0.4933 (7)	0.3734 (3)	0.0361 (14)	
H4	0.056199	0.474885	0.331615	0.043*	
C5	0.1132 (8)	0.3943 (6)	0.4210 (3)	0.0314 (14)	
H5	0.066424	0.308183	0.410259	0.038*	
C6	0.3360 (7)	0.7797 (5)	0.4701 (2)	0.0186 (11)	
C7	0.7871 (9)	0.8618 (7)	0.7123 (3)	0.0367 (15)	
C8	0.6168 (8)	0.8574 (6)	0.6809 (3)	0.0258 (12)	
C9	0.4876 (9)	0.7953 (8)	0.7142 (3)	0.0402 (16)	
H9	0.370816	0.793989	0.696302	0.048*	
C10	0.5309 (12)	0.7351 (9)	0.7740 (4)	0.054 (2)	
H10	0.445567	0.689641	0.796168	0.065*	
C11	0.7019 (13)	0.7440 (9)	0.7997 (3)	0.058 (2)	
H11A	0.730394	0.704053	0.840298	0.069*	
C12	0.5703 (7)	0.9133 (5)	0.6139 (2)	0.0211 (11)	

### Atomic displacement parameters (Å^2^)

**Table:**

	*U*^11^	*U*^22^	*U*^33^	*U*^12^	*U*^13^	*U*^23^
Cu1	0.0134 (3)	0.0128 (3)	0.0144 (3)	0.0004 (2)	0.0001 (2)	0.0006 (2)
Br1	0.0477 (4)	0.0450 (4)	0.0450 (4)	−0.0018 (3)	−0.0018 (3)	0.0041 (3)
Br2	0.0700 (7)	0.1137 (9)	0.0637 (6)	−0.0456 (6)	−0.0206 (5)	0.0186 (6)
N1	0.019 (2)	0.014 (2)	0.045 (3)	0.0007 (18)	0.003 (2)	0.000 (2)
N2	0.066 (4)	0.057 (4)	0.029 (3)	0.007 (3)	−0.021 (3)	0.008 (3)
O1	0.0135 (17)	0.0172 (18)	0.030 (2)	0.0008 (14)	0.0027 (15)	−0.0025 (16)
O2	0.0172 (19)	0.0185 (19)	0.040 (2)	−0.0033 (14)	0.0033 (16)	−0.0058 (16)
O3	0.0155 (18)	0.0173 (18)	0.037 (2)	−0.0026 (14)	0.0034 (16)	−0.0043 (16)
O4	0.031 (2)	0.033 (2)	0.0157 (18)	0.0089 (17)	−0.0009 (16)	0.0044 (16)
O5	0.032 (2)	0.033 (2)	0.0184 (19)	0.0068 (18)	−0.0004 (16)	0.0049 (16)
C1	0.014 (2)	0.017 (3)	0.033 (3)	0.001 (2)	0.003 (2)	−0.001 (2)
C2	0.015 (2)	0.014 (2)	0.029 (3)	−0.0006 (19)	0.001 (2)	−0.001 (2)
C3	0.037 (3)	0.023 (3)	0.034 (3)	−0.005 (2)	−0.007 (3)	0.004 (2)
C4	0.036 (3)	0.032 (3)	0.038 (3)	−0.005 (3)	−0.011 (3)	−0.009 (3)
C5	0.024 (3)	0.017 (3)	0.051 (4)	−0.002 (2)	−0.005 (3)	−0.008 (3)
C6	0.018 (3)	0.016 (3)	0.021 (3)	−0.003 (2)	−0.003 (2)	0.003 (2)
C7	0.048 (4)	0.038 (3)	0.023 (3)	−0.004 (3)	−0.008 (3)	0.001 (3)
C8	0.037 (3)	0.025 (3)	0.015 (3)	0.005 (2)	−0.003 (2)	0.005 (2)
C9	0.045 (4)	0.048 (4)	0.027 (3)	0.003 (3)	0.005 (3)	0.012 (3)
C10	0.071 (5)	0.059 (5)	0.034 (4)	0.010 (4)	0.015 (4)	0.022 (3)
C11	0.089 (6)	0.060 (5)	0.024 (3)	0.007 (5)	0.002 (4)	0.023 (3)
C12	0.031 (3)	0.015 (2)	0.017 (3)	−0.001 (2)	−0.003 (2)	0.000 (2)

### Geometric parameters (Å, º)

**Table:**

Cu1—O5^i^	1.950 (4)	O5—C12	1.261 (7)
Cu1—O4	1.958 (4)	C1—C2	1.386 (8)
Cu1—O3^i^	1.978 (4)	C2—C3	1.381 (8)
Cu1—O2	1.979 (4)	C2—C6	1.499 (7)
Cu1—O1	2.120 (4)	C3—C4	1.384 (9)
Cu1—Cu1^i^	2.6470 (11)	C3—H3	0.9300
Br1—C1	1.875 (6)	C4—C5	1.371 (9)
Br2—C7	1.870 (7)	C4—H4	0.9300
N1—C1	1.314 (7)	C5—H5	0.9300
N1—C5	1.334 (8)	C7—C8	1.396 (9)
N2—C7	1.326 (8)	C8—C9	1.374 (9)
N2—C11	1.331 (11)	C8—C12	1.496 (7)
O1—H11	0.815 (10)	C9—C10	1.378 (9)
O1—H12	0.815 (10)	C9—H9	0.9300
O2—C6	1.245 (6)	C10—C11	1.362 (12)
O3—C6	1.254 (6)	C10—H10	0.9300
O4—C12	1.239 (7)	C11—H11A	0.9300
			
O5^i^—Cu1—O4	167.76 (17)	C2—C3—C4	119.2 (6)
O5^i^—Cu1—O3^i^	89.64 (17)	C2—C3—H3	120.4
O4—Cu1—O3^i^	89.35 (17)	C4—C3—H3	120.4
O5^i^—Cu1—O2	88.95 (17)	C5—C4—C3	118.3 (6)
O4—Cu1—O2	89.47 (17)	C5—C4—H4	120.9
O3^i^—Cu1—O2	167.80 (15)	C3—C4—H4	120.9
O5^i^—Cu1—O1	98.06 (16)	N1—C5—C4	123.6 (5)
O4—Cu1—O1	94.05 (15)	N1—C5—H5	118.2
O3^i^—Cu1—O1	103.05 (15)	C4—C5—H5	118.2
O2—Cu1—O1	89.15 (15)	O2—C6—O3	126.2 (5)
O5^i^—Cu1—Cu1^i^	87.08 (12)	O2—C6—C2	116.3 (5)
O4—Cu1—Cu1^i^	80.69 (12)	O3—C6—C2	117.5 (4)
O3^i^—Cu1—Cu1^i^	87.92 (11)	N2—C7—C8	124.0 (6)
O2—Cu1—Cu1^i^	79.91 (11)	N2—C7—Br2	114.0 (5)
O1—Cu1—Cu1^i^	167.85 (11)	C8—C7—Br2	121.9 (5)
C1—N1—C5	117.1 (5)	C9—C8—C7	116.7 (5)
C7—N2—C11	117.0 (7)	C9—C8—C12	119.4 (5)
Cu1—O1—H11	118 (4)	C7—C8—C12	123.9 (5)
Cu1—O1—H12	105 (4)	C8—C9—C10	120.1 (7)
H11—O1—H12	107 (4)	C8—C9—H9	120.0
C6—O2—Cu1	127.8 (3)	C10—C9—H9	120.0
C6—O3—Cu1^i^	118.1 (3)	C11—C10—C9	118.3 (7)
C12—O4—Cu1	126.8 (3)	C11—C10—H10	120.9
C12—O5—Cu1^i^	119.1 (3)	C9—C10—H10	120.9
N1—C1—C2	124.5 (5)	N2—C11—C10	123.9 (6)
N1—C1—Br1	116.2 (4)	N2—C11—H11A	118.1
C2—C1—Br1	119.3 (4)	C10—C11—H11A	118.1
C3—C2—C1	117.3 (5)	O4—C12—O5	126.3 (5)
C3—C2—C6	120.9 (5)	O4—C12—C8	117.6 (5)
C1—C2—C6	121.8 (5)	O5—C12—C8	116.0 (5)
			
C5—N1—C1—C2	1.0 (8)	C11—N2—C7—C8	−0.5 (11)
C5—N1—C1—Br1	−178.0 (4)	C11—N2—C7—Br2	176.5 (6)
N1—C1—C2—C3	0.0 (8)	N2—C7—C8—C9	2.4 (10)
Br1—C1—C2—C3	179.0 (4)	Br2—C7—C8—C9	−174.4 (5)
N1—C1—C2—C6	−178.5 (5)	N2—C7—C8—C12	−176.5 (6)
Br1—C1—C2—C6	0.5 (7)	Br2—C7—C8—C12	6.7 (9)
C1—C2—C3—C4	−1.0 (9)	C7—C8—C9—C10	−3.3 (10)
C6—C2—C3—C4	177.6 (6)	C12—C8—C9—C10	175.6 (6)
C2—C3—C4—C5	0.9 (10)	C8—C9—C10—C11	2.6 (12)
C1—N1—C5—C4	−1.1 (9)	C7—N2—C11—C10	−0.4 (13)
C3—C4—C5—N1	0.2 (10)	C9—C10—C11—N2	−0.7 (14)
Cu1—O2—C6—O3	3.4 (8)	Cu1—O4—C12—O5	2.3 (8)
Cu1—O2—C6—C2	−177.5 (3)	Cu1—O4—C12—C8	179.3 (4)
Cu1^i^—O3—C6—O2	−2.0 (7)	Cu1^i^—O5—C12—O4	−2.9 (8)
Cu1^i^—O3—C6—C2	178.9 (3)	Cu1^i^—O5—C12—C8	−179.9 (4)
C3—C2—C6—O2	−87.8 (6)	C9—C8—C12—O4	−138.3 (6)
C1—C2—C6—O2	90.7 (6)	C7—C8—C12—O4	40.6 (8)
C3—C2—C6—O3	91.3 (6)	C9—C8—C12—O5	39.0 (8)
C1—C2—C6—O3	−90.2 (6)	C7—C8—C12—O5	−142.2 (6)

Symmetry code: (i) −*x*+1, −*y*+2, −*z*+1.

### Hydrogen-bond geometry (Å, º)

**Table:**

*D*—H···*A*	*D*—H	H···*A*	*D*···*A*	*D*—H···*A*
O1—H11···O3^ii^	0.82 (1)	2.07 (2)	2.868 (5)	165 (6)
O1—H12···N1^iii^	0.82 (1)	2.02 (2)	2.816 (6)	166 (6)
C11—H11*A*···O1^iv^	0.93	2.51	3.403 (8)	161

Symmetry codes: (ii) *x*+1, *y*, *z*; (iii) −*x*+1, −*y*+1, −*z*+1; (iv) *x*, −*y*+3/2, *z*+1/2.
