# Supplementary material for: The first coordination compound of deprotonated 2-bromo­nicotinic acid: crystal structure of a dinuclear paddle-wheel copper(II) complex
Source: Acta Crystallogr E Crystallogr Commun. 2020 Jan 17;76(Pt 2):225–30. doi: 10.1107/S2056989020000390 (PMC7001845; doi:10.1107/S2056989020000390)
Supplement: Supplementary file 3 [file e-76-00225-sup3.docx]

The first coordination compound of 2-bromo­nicotinic acid. Crystal structure of a dinuclear paddle-wheel copper(II) cluster with 2-bromo­nicotinic acid.

**Nives Politeo,**^a^ **Mateja Pisa**č**i**ć**,**^b^ **Marijana** Đ**akovi**ć**,**^b^ **Vesna Sokol**^a^*** and Boris-Marko Kukovec**^a^

**^a^**Department of Physical Chemistry, Faculty of Chemistry and Technology, University of Split, Ruđera Boškovića 35, HR-21000 Split, Croatia, and **^b^**Department of Chemistry, Faculty of Science, University of Zagreb, Horvatovac, 102a, HR-10000 Zagreb, Croatia

Correspondence email: vsokol@ktf-split.hr

**Click the grey 'Authors' label above or use the 'IUCr authors' toolbar button to edit the authors**

Supporting information


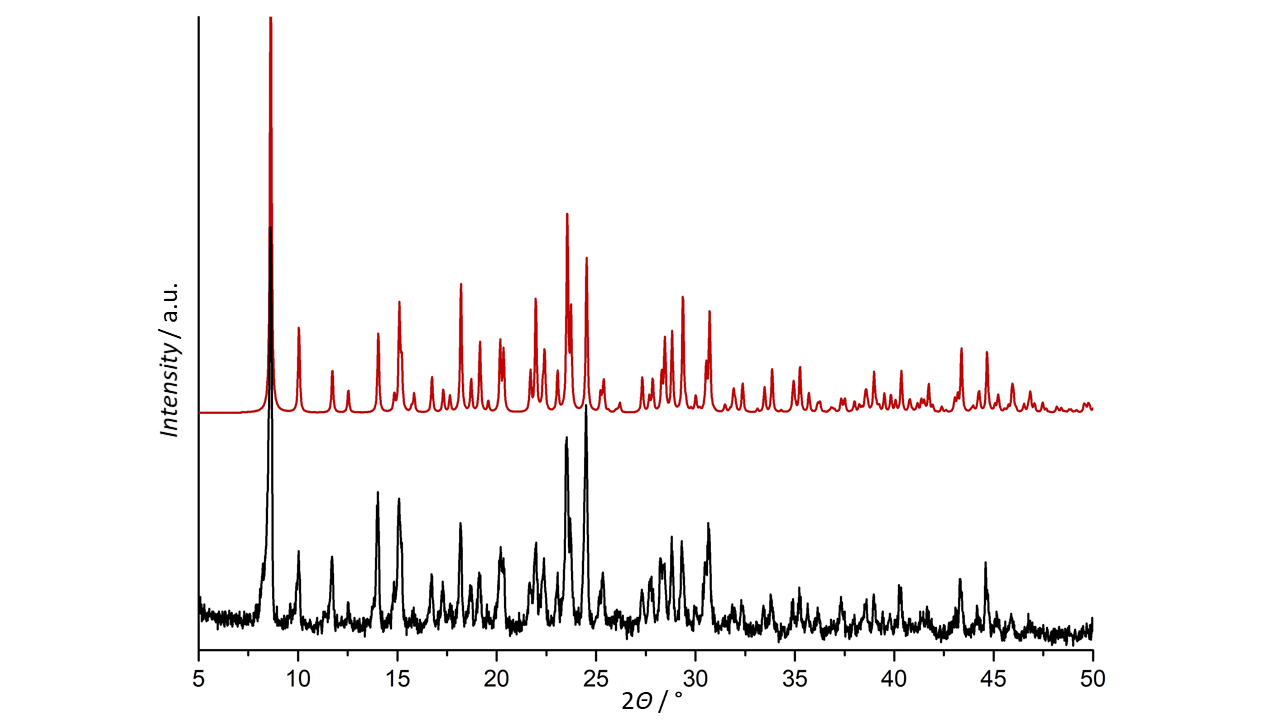


1. Experimental (bottom) and calculated (top) PXRD traces of **1**.


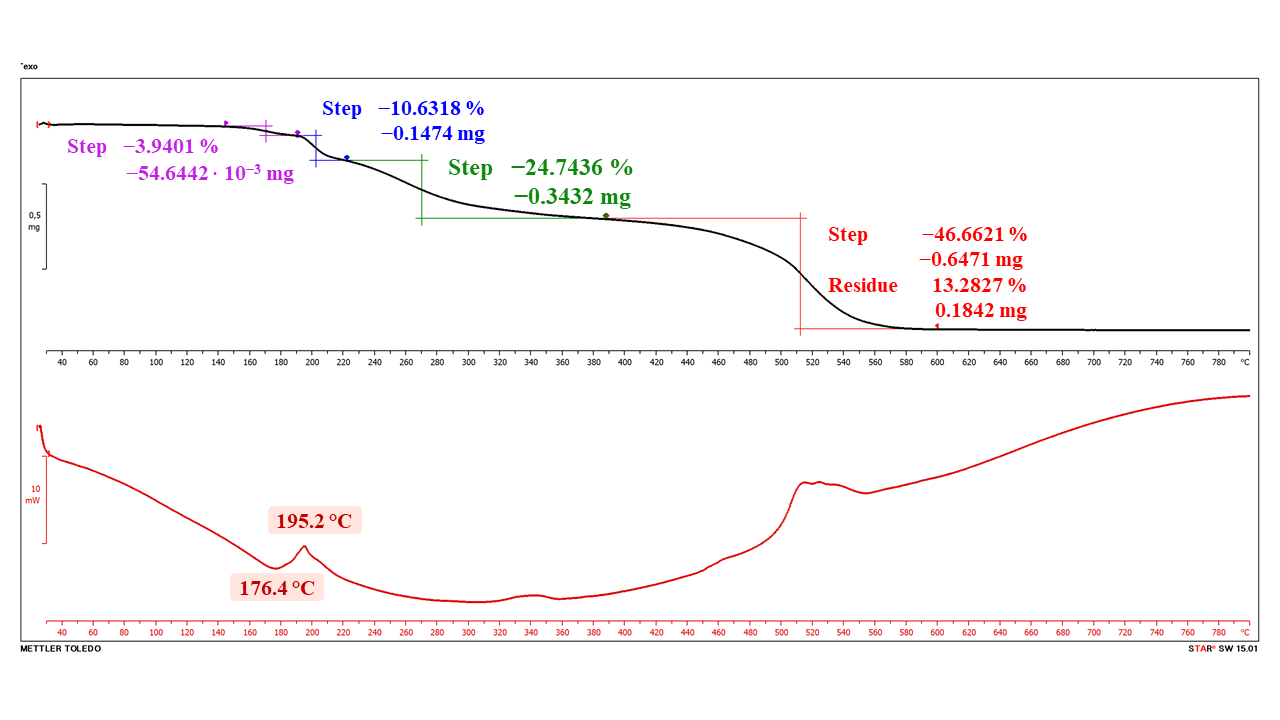


1. TGA (top) and DSC (bottom) curves of **1**.


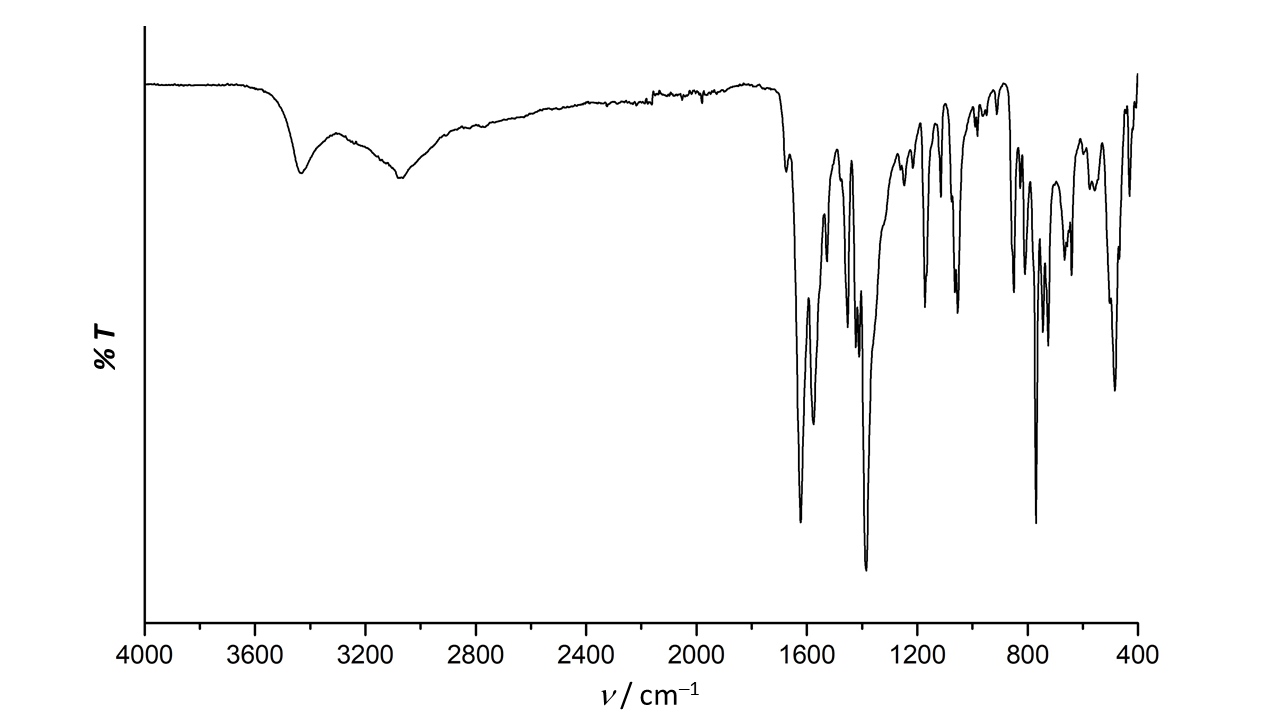


1. IR spectrum of **1**.

**Table S1** IR bands of **1**.

| *ν* (cm^−1^) | %*T* |
| --- | --- |
| 3432 | 96 |
| 3082 | 96 |
| 3075 | 96 |
| 3065 | 96 |
| 1623 | 83 |
| 1576 | 87 |
| 1452 | 90 |
| 1411 | 89 |
| 1385 | 82 |
| 1172 | 91 |
| 1115 | 95 |
| 1054 | 91 |
| 850 | 91 |
| 810 | 91 |
| 770 | 82 |
| 745 | 89 |
| 726 | 88 |
| 641 | 90 |
| 484 | 85 |
| 431 | 92 |
